# A Review of Hybrid Fiber-Optic Distributed Simultaneous Vibration and Temperature Sensing Technology and Its Geophysical Applications

**DOI:** 10.3390/s17112511

**Published:** 2017-11-01

**Authors:** Khalid Miah, David K. Potter

**Affiliations:** 1 Geophysical Engineering Department, Montana Tech of the University of Montana, Butte, MT 59701, USA; 2 Physics Department, University of Alberta, Edmonton, AB T6G2E1, Canada; dkpotter@ualberta.ca

**Keywords:** fiber-optic distributed sensing, vibration, temperature, geophysical applications, digital signal processing

## Abstract

Distributed sensing systems can transform an optical fiber cable into an array of sensors, allowing users to detect and monitor multiple physical parameters such as temperature, vibration and strain with fine spatial and temporal resolution over a long distance. Fiber-optic distributed acoustic sensing (DAS) and distributed temperature sensing (DTS) systems have been developed for various applications with varied spatial resolution, and spectral and sensing range. Rayleigh scattering-based phase optical time domain reflectometry (OTDR) for vibration and Raman/Brillouin scattering-based OTDR for temperature and strain measurements have been developed over the past two decades. The key challenge has been to find a methodology that would enable the physical parameters to be determined at any point along the sensing fiber with high sensitivity and spatial resolution, yet within acceptable frequency range for dynamic vibration, and temperature detection. There are many applications, especially in geophysical and mining engineering where simultaneous measurements of vibration and temperature are essential. In this article, recent developments of different hybrid systems for simultaneous vibration, temperature and strain measurements are analyzed based on their operation principles and performance. Then, challenges and limitations of the systems are highlighted for geophysical applications.

## 1. Introduction

This paper discusses fundamentals, operation principles, known limitations and geophysical applications of hybrid fiber-optic multi-parameter detection systems, developed in recent years. Fiber-optic sensing technology (FOS) has the potential to replace conventional electromechanical-based temperature and vibration sensors used in civil, environmental, mining, and energy exploration, especially in harsh and difficult-to-access environments. Fiber-optic cable is resilient to electromagnetic interference and can be used for applications in harsh (high temperature and pressure) environments. At the same time, the transmitted light pulses through the fiber are very sensitive to ambient conditions, such as the temperature, strain and vibration. These characteristics of the optical fiber make it useful as a collection of sensors for measuring surrounding temperature and dynamic vibration with fine spatial and temporal resolution.

Due to great efforts from researchers in the fiber-optic sensing community over the last 10 years, the performance of distributed acoustic sensing (DAS) and distributed temperature sensing (DTS) systems has improved for certain applications requiring large area coverage with high location accuracy. However, real-time applications, poor signal-to-noise ratio, coupling of the fiber to the medium, and handling large data sets are the bottleneck in taking full advantage of the fiber-optic sensing technology. Simultaneous measurements of temperature and vibration will eliminate the need for two separate systems (DAS and DTS), thus improving measurement efficiency and reducing overall cost.

Distributed vibration and temperature sensing (DVTS) is a passive fiber-optic sensor technology that can detect both acoustic field and temperature along the length of the fiber. Temporally continuous measurements can be made along the fiber length with high-frequency response and fine spatial resolution. Several commercial distributed fiber-optic sensing systems have been developed by industry for measuring temperature or vibration, but not both simultaneously. However, the systems are very application specific.

Distributed sensing systems can transform an optical fiber cable into an array of virtual sensing devices, allowing users to detect and monitor both temperature and vibration near the cable. The challenge has been to find a mechanism that would allow the key structural parameters to be determined at any point along a fiber-optic cable with high sensitivity and spatial resolution, and yet within acceptable temporal resolution for dynamic vibration, strain and temperature detection.

Distributed fiber-optic sensing systems have the potential to become one of the core technologies in collecting dynamic in situ information (strain and temperature) of various structures as a function of spatial distribution of the monitoring probe. Thus, these types of sensing systems can be combined with emerging instrumentation technology to assist people in making decisions on the safety of personnel and structures. This can also form a real-time link between the local monitoring probe and decision makers through the Internet using telecommunication devices. The real-time information on vibration and temperature can provide early warning, helping officials reduce potential civil structure failure along with loss of lives and injury.

Applications of fiber-optic DAS (distributed acoustic sensing) and DTS (distributed temperature sensing) systems have exploded in the past five years, especially in the oil and gas industry due to considerable investment in research and development [1]. There are many applications of DAS and DTS systems in the energy industry, some are successfully field-tested and others are yet to be tested. The field-tested applications include hydraulic fracture monitoring, vertical seismic profiling, gas-lift-optimization, flow profiling, and sand detection [2,3,4,5,6,7,8]. Some applications that may be feasible with the existing DAS and DTS systems include gas breakthrough detection and electrical submersible pump (ESP) monitoring in the oilfield. Even though fiber-optic DAS systems of varying capabilities have been developed for natural resource explorations in recent years, know-how of the underlying technology required to reproduce the systems and advance the technology is not available to researchers outside the industry because of proprietary information and patent constraints. Also, no viable fiber-optic system for simultaneous dynamic measurements of vibration and temperature exists beyond a few published articles [9,10]. Thus, the rationale for designing, building and testing a low-cost hybrid fiber-optic DVTS system is three-fold: providing a cost-efficient alternative for simultaneous measurement of vibration and temperature on a par with commercial systems; advancing the fiber-optic sensing technology, thus opening doors for new applications; and sharing the underlying technology and expertise with a larger research community. This review paper is an effort to summarize recent developments in distributed fiber-optic sensing, especially for multi-parameter (temperature, vibration, and strain) detection, and highlight challenges and limitations of such systems in geophysical applications. Fiber-optic cables and principle of light propagation is discussed in Section 2. Distributed sensing systems based on interferometry, Rayleigh or Brillouin scattering technology for temperature and vibration measurements are summarized in Section 3. Experimental setup and key parameters of a recently developed hybrid system for simultaneous vibration and temperature measurements are discussed in Section 4. Two main challenges of distributed measurements: FOS-specific fiber design and sensor-medium coupling are noted in Section 5. The importance of multi-parameter detection in geophysical engineering is highlighted in Section 6. Challenges associated with geophysical applications are discussed in Section 7. Finally, Section 8 concludes this article.

## 2. Fiber-Optic Cable and Light Scattering Basics

In distributed fiber-optic sensing, the fiber optical cable itself acts as a continuous array of sensors. As an incident light propagates through the fiber core, any disturbance due to the change in physical parameters (temperature, vibration and strain) affects the length, diameter, and refractive index of the fiber core. These effects cause the incident light to backscatter, and then be detected at the receiver end. Intensity, phase shift, and frequency shift of the backscattered signal are interpreted for location and amplitude of the physical parameters along the length of fiber. Light attenuation loss, mode excitation and scatterings are directly related to the type of fiber used. Core diameter, materials used in the core and cladding, and materials used for the protective sheath, all affect the overall sensitivity of the sensing system.

### 2.1. Fiber-Optic Cable

**Figure 1:**
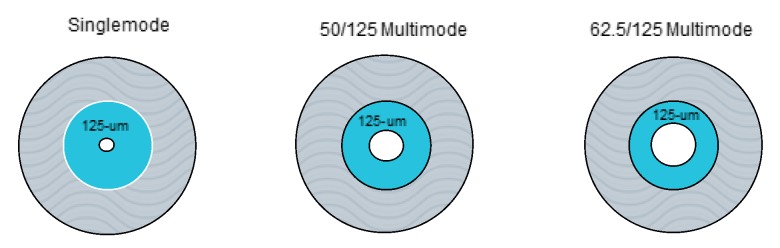
Schematic of optical fiber based on the core diameter and mode.

Advances in optical fiber design and digital signal processing have been pushing the data transmission rate beyond terabits per second [11]. Most telecommunication fibers can be either singlemode or multimode fibers, where each consists of a light propagating core surrounded by cladding with different refractive index. The fiber is then surrounded by a protective sheath to protect it from the element (Figure 1).

**Figure 2:**
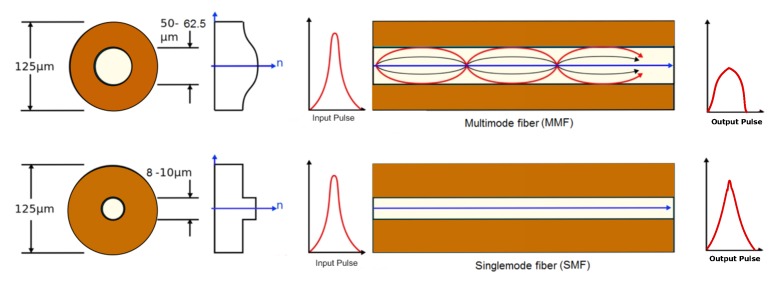
Schematic of light propagation in a singlemode versus multimode fiber (adapted from [13]).

One of the limitations in achieving a longer sensing range is the inherent attenuation loss along the fiber length. Attenuation of light pulses through the fiber depends on the core diameter and wavelength of the passing light [12]. Singlemode fibers are preferable for distributed sensing over long distance due to less attenuation compared to multimode fibers. Attenuation loss depends on the wavelength. It is found that the loss is minimum (0.2 dB/km) at 1550 nm wavelength in a singlemode fiber. Table 1 shows attenuation loss of different types of fibers. A schematic displaying propagation of a laser pulse through a singlemode versus multimode fiber is shown in Figure 2. Intrinsic attenuation loss is higher in a multimode fiber than the singlemode fiber of the same length.

### 2.2. Light Scattering

**Figure 3:**
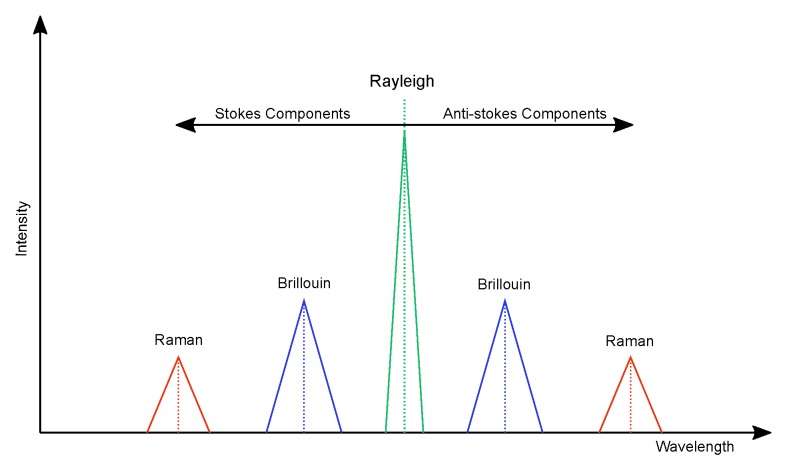
Schematic displaying Rayleigh, Raman, and Brillouin peaks in the electromagnetic spectrum. Frequency increases to the right; and wavelength to the left. (Adapted from [15].)

The optical fiber cable is sensitive to changes in the surrounding environment (such as temperature, strain, and stress). To measure these changes, distributed sensors monitor the backscattered light along the length of the fiber. The backscattered trace is continuous in time, where each point in time corresponds to a particular location along the fiber. This time information can be converted into distance by using the speed of light in the fiber and length of the fiber [14]. The spectrum of backscattered light shows three types of scattering: Rayleigh, Brillouin, and Raman (Figure 3) [15]. In the electromagnetic spectrum, light scattering in a fiber-optic cable can be separated into three components: Rayleigh, Stokes and Anti-Stokes. Rayleigh scattering is directly correlated to the laser source wavelength and very sensitive to strains induced in the fiber due to external vibration. In the Stokes band, the Brillouin scattering is strain-independent while the Raman scattering is temperature-independent. In the Anti-Stokes band, the Brillouin scattering is strain-dependent while the Raman scattering is temperature-dependent [16].

**Figure 4:**
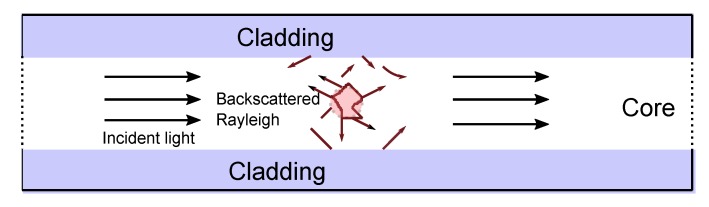
Schematic of a spontaneous Rayleigh backscattering process through the core of an optical fiber cable. (Adapted from [19].)

Rayleigh scattering is an elastic process due to randomly-occurring inhomogeneities in the refractive index of the fiber core. No energy is transfered in the glass, so the frequency of the Rayleigh scattering is the same as the incident light pulse. The Rayleigh backscattered light has a time delay, used for spatially distributed sensing (Figure 4) along the fiber length. In general, Rayleigh scattering provides a temperature and strain-invariant reference attenuation distribution, useful in sensing those parameters along the fiber using either Raman or Brillouin scattering [1,17,18].

Brillouin scattering involves interactions among incident wave, scattered wave and phonons. These interactions result in very a small frequency shift (approximately 11 GHz at 1530 nm). For a given incident light, this frequency shift solely depends on the acoustic velocity and fiber refractive index, which depends on both the intrinsic characteristics (e.g., fiber core composition) and environment variables (e.g., temperature and strain). In typical backscattering, the Brillouin signal is about 15–20 dB weaker than the Rayleigh signal. In Raman scattering, the incident photon is scattered by a molecule within the core material, which simultaneously undergoes a two-state transition process, and can either produce or absorb phonons. Like Brillouin scattering, the Raman scattering causes a frequency shift (approximately 13.0 THz at 1550 nm) in the scattered wave. Both the elastic property of Rayleigh scattering and the inelastic property of Brillouin scattering can be utilized for simultaneous measurements of temperature and dynamic vibration in a distributed sensing system.

There are several advantages of using the Brillouin scattering over Raman as a basis for distributed temperature sensing. Despite having a smaller frequency shift ( 11 GHz), the Brillouin gain bandwidth makes it an ideal candidate for natural electrical separation in heterodyne systems. Considering a small frequency shift, both the incident and Brillouin wavelength experience almost identical attenuation. So, with the 1550 nm light source, the spatial sensing range can be increased by keeping the loss at a minimum. Also Brillouin signals are an order of magnitude stronger than the Raman, thus having a higher signal-to-noise ratio (SNR) and allowing improvement in sensor spatial resolution and sensing range. Even though Brillouin scattering is capable of sensing both temperature and strain, light attenuation along the fiber is much greater than the Rayleigh, and thus not feasible in dynamic strain measurement. So a combination of Rayleigh and Brillouin scattering can be used for simultaneous measurements of vibration and temperature on singlemode fiber with a 1550 nm wavelength light source.

Design emphasis for a multi-parameter detection system will be on dynamic vibration measurement rather than on temperature, especially for geophysical exploration applications where the ground vibration signature is used for subsurface rock characterization. It can be noted that Raman scattering would be an ideal choice for distributed sensing, if temperature is the only parameter of interest, since Raman scattering based DTS systems have higher temperature sensitivity than that of Brillouin scattering-based systems.

## 3. Fiber-Optic Sensing (FOS) Technology

In standard optical time domain reflectometry (OTDR), a broadband noncoherent light source is used to detect anomalies along the length of optical fiber by analyzing the intensity of the Rayleigh backscattered signal [20]. However, phase information of the returned light is not available in the standard OTDR. In general, FOS technology for distributed parameter detection spans from interferometry-based systems to Rayleigh and/or Brillouin scattering-based phase-OTDR and polarization-OTDR systems, using either coherent or direct detection methods [15,21,22].

### 3.1. Interferometry-Based Sensing Method

**Figure 5:**
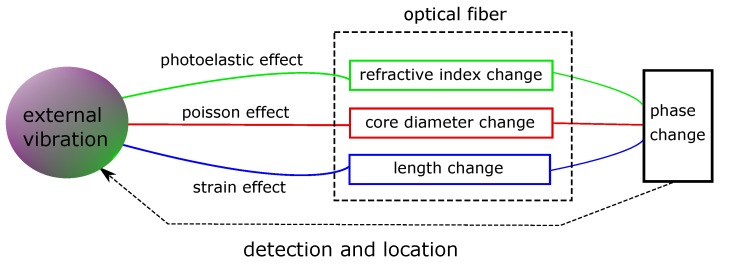
Schematic of the phase change detection due to external vibration using interferometric-based fiber-optic sensing. (Adapted from [17].)

In recent distributed fiber-optic vibration sensing systems, some combinations of interferometric- sensing and backscattered-based sensing technology are used. For interferometry-based distributed sensing, local vibration information is acquired on the basis of dynamic phase change of the optical wave [17]. This phase change is directly related to changes in the fiber length, refractive index of the core, and diameter of the core caused by strain, photoelastic and Poisson effects resulting from any external vibration (Figure 5). In general, when light passes through a single mode fiber of length *L* at a speed of *v*, the phase delay at the other end can be expressed as [17]:

(1) ϕ=βL

where β is the wave propagation constant. Now, if the fiber is subject to an external pressure *P*, then the change in the phase delay can be expressed as [17]:

(2) $$\Delta \phi =\frac{\beta LP}{E\left(1-2\nu \right)[1/2n^{2}\left(P_{1}+2P_{2}\right)-1]}$$

where *E* is the Young’s modulus, ν is the Poisson’s ratio, *n* is the fiber refractive index, and $P_{1}$ and $P_{2}$ are pressures associated with *P*. This phase change is proportional to the external pressure *P* assuming the other parameters remain constant.

In general, interferometric-based distributed vibration sensing technology includes Sagnac, Fabry–Perot, Mach–Zehnder Interferometer (MZI) and Michelson Interferometer (MI) systems [23,24].

**Figure 6:**
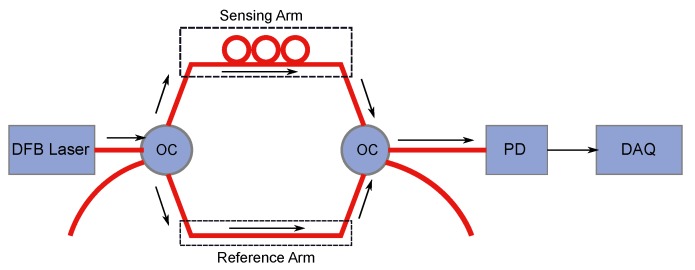
Schematic of a simple Mach–Zehnder Interferometer (MZI)-based detection system. DFB-Laser: Distributed Feedback-Laser, PC: Polarization Controller, OC: Optical Coupler, PD: Photodiode, and DAQ: Data Acquisition. (Adapted from [25].)

However, the MZI sensor technology is widely used in conjunction with the Rayleigh backscattered-based sensing for vibration measurement owing to its simple structure and straightforward location detection method. In a basic MZI-based sensing, the light is split into two separate paths using a fiber-to-fiber coupler. One path leads into the sensing fiber and the other one into the vibration-shielded reference fiber. The return light beams are then recombined using a second fiber-to-fiber coupler and the phase shift is measured through a high-speed photodetector (PD) (Figure 6). This phase shift results from changes in the length and refractive index of the sensing fiber core, caused by external vibration [23]. For practical purposes, a dual MZI (DMZI) configuration is widely used for vibration sensing since it can be used to detect both the signal and its [17] location at the same time, unlike a single MZI configuration.

### 3.2. Rayleigh and Brillouin Scattering-Based Sensing Methods

**Figure 7:**
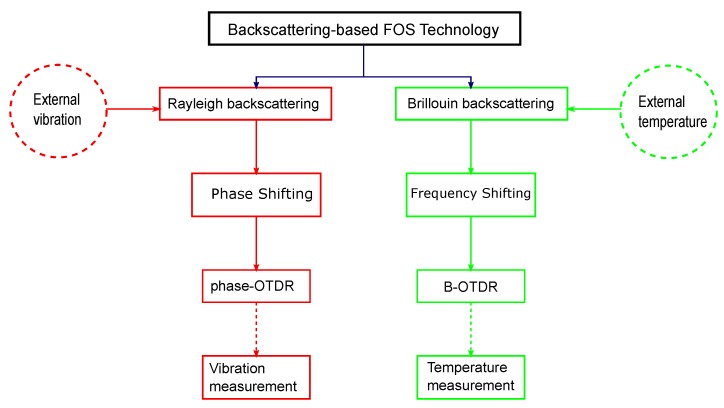
Flow chart displaying the ϕ-OTDR and B-OTDR scattering techniques for vibration and temperature measurements.

Rayleigh scattering is an elastic process and can be used for vibration measurement using standard telecommunication fibers. Brillouin scattering is an inelastic process and ideal for temperature and strain measurements. Even though Brillouin backscattered signals are about 15–20 dB weaker than the Rayleigh scattering, both scatterings can be used as a basis for simultaneous measurements of vibration and temperature in a distributed fiber-optic sensing system [26,27,28]. A combination of Phase-OTDR (ϕ-OTDR) with a Mach–Zehnder Interferometer (MZI) and Brillouin-OTDR (B-OTDR) can be used with a coherent narrow laser source for simultaneous measurements of vibration and temperature. Laser pulses of various width and intensity can be encoded to form a pulse-train and injected into the sensing fiber repeatedly for dynamic vibration and temperature measurements. A flow diagram displaying a ϕ-OTDR for vibration and B-OTDR for temperature measurements is shown in Figure 7.

However, Rayleigh–Brillouin scattering-based sensing technology is more robust and easier to implement in a singlemode fiber for simultaneous measurements of vibration and temperature [20,22,29,30]. Both Rayleigh and Brillouin scattering-based sensing systems measure changes in the phase, polarization, and frequency of the propagating light waves utilizing some variations of the optical time domain reflectometry (OTDR) technology. In an OTDR system, the location of a reflection point is determined by the time delay between the launched light pulse and the corresponding Rayleigh backscattered signal, and can be expressed as [17]:

(3) $$x=\frac{1}{2n_{g}}c\tau$$

where τ is the time delay, *c* is the light propagation speed in the fiber, $n_{g}$ is the group refractive index of the fiber, and *x* is the distance of the reflection point from the reference. Spatial resolution, Δx of the measurement can be expressed as:

(4) $$\Delta x=\frac{1}{2n_{g}}cT_{P}$$

where $T_{P}$ is the pulse width. The power of the backscattered signal at a distance *l* from the reference can be calculated as:

(5) $$P_{s}=\frac{1}{2}F\times \alpha_{s}\times v_{g}\times \tau \times P_{i}\times e^{-2\alpha l}$$

where *F* is the capture coefficient, $\alpha_{s}$ is the Rayleigh scattering coefficient, $v_{g}$ is the group velocity in the fiber, $P_{i}$ is the input power, and α is the fiber loss coefficient.

#### 3.2.1. Rayleigh Scattering-Based ϕ- OTDR for Vibration Sensing

In a direct detection ϕ-OTDR system, a highly coherent laser pulse-train is repeatedly injected in the sensing fiber, and the reflected Rayleigh backscattered signal is acquired to form ϕ-OTDR traces [28,31]. In general, each trace is a plot of optical power versus time, where each point indicates the location and energy level of the event along the fiber length [32]. The spatial resolution of an OTDR-based system depends on the pulse width in optical domain, while the detector bandwidth dictates the resolution in the digital domain [1]. The digital signal is then acquired and saved for post-processing using a high-speed data acquisition device (DAQ). The quality of the signal acquired depends largely on the dynamic range and sampling rate of the DAQ system. Usually, a DAQ system with either a single- or dual-channel capability with a sampling rate in the order of Gigahertz (GHz) frequencies is used in a distributed optical sensing application. In some distributed fiber-optic sensing systems, a dual-channel DAQ is used to save the reference and scattered signals separately. The laser pulse-train repetition rate and light source output power dictate the temporal resolution and linear sensing range. In theory, increasing the laser output power and shortening the pulse width can increase overall sensing range and spatial resolution, respectively. However, continuous sensing over a long distance comes at a cost of dealing with nonlinear noise effects and spurious scatterings (both spontaneous and stimulating). Over the last decade, several distributed ϕ-OTDR-based vibration detection systems, focused on particular applications, have been proposed and implemented with varied levels of performance and complexity [21,33]. In a recent publication, Wang et al. [34] have experimentally implemented ϕ-OTDR for real-time 1-D and 2-D vibration measurements using the differential method and the Prewitt edge detection method, respectively.

**Figure 8:**
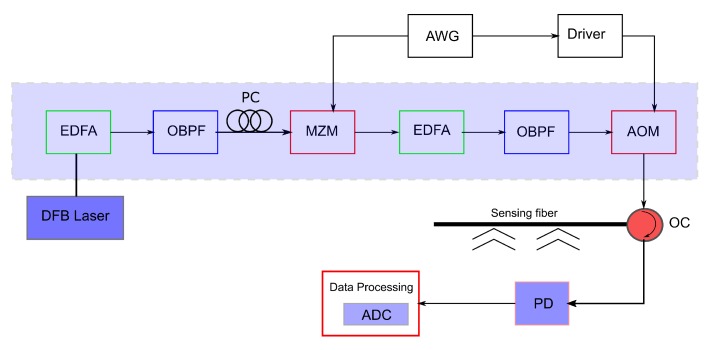
Experimental setup of a pulse-coded standard ϕ-OTDR system. DFB: Distributed Feedback, AOM: Acousto-Optic Modulator, EDFA: Erbium-Doped Fiber Amplifier, MZM: Mach–Zehnder Modulator, OBPF: Optical Bandpass Filter, PC: Polarization Controller, AWG: Arbitrary Waveform Generator, OC: Optical Coupler, PD: Photodetector, and ADC: Analog-Digital Converter [35].

Recently, Muanenda et al. [35] proposed and implemented a cost-effective direct detection pulse coded ϕ-OTDR with MZI for distributed dynamic vibration measurement. Their system is capable of measuring vibrations of up to 500 Hz with 5-m spatial resolution over a sensing distance of 5 km on a singlemode fiber. A linear Simplex pulse encoding technique [36] is used to design an optimal pulse-train for vibration sensing. It is shown that an effective pulse coding scheme can improve the sensitivity of an OTDR-based system [2,32,36]. The experimental setup of a pulse-coded direct detection ϕ-OTDR vibration sensing system with pulse coding is shown in Figure 8 [35].

In Section 4, a similar ϕ-OTDR technique is integrated with a Brillouin-OTDR technique for simultaneous measurements of vibration and temperature.

#### 3.2.2. Brillouin Scattering-Based OTDR for Temperature Sensing

Distributed temperature measurement using an optical fiber as the sensing element exploits either the Brillouin or Raman scattering. In comparison to Raman scattering, Brillouin scattering-based distributed temperature sensing (DTS) is characterized by higher backscattered intensities and is resilient to wavelength-dependent loss. However, this approach of distributed temperature measurement requires complex processing at the receiver end. In the last two decades, Brillouin scattering-based distributed fiber-optic sensors were investigated for temperature and strain measurements with fine spatial resolution and longer sensing range. The principle of distributed Brillouin scattering sensors for temperature measurement is based on the change in mean density associated with the velocity of sound, and change in the refractive index of the fiber core, which causes a Brillouin frequency shift; these changes are directly related to the surrounding temperature variations [1]. Hence, Brillouin scattering-based temperature sensing involves measuring the frequency shift of the Brillouin peaks induced by temperature variations.

**Figure 9:**
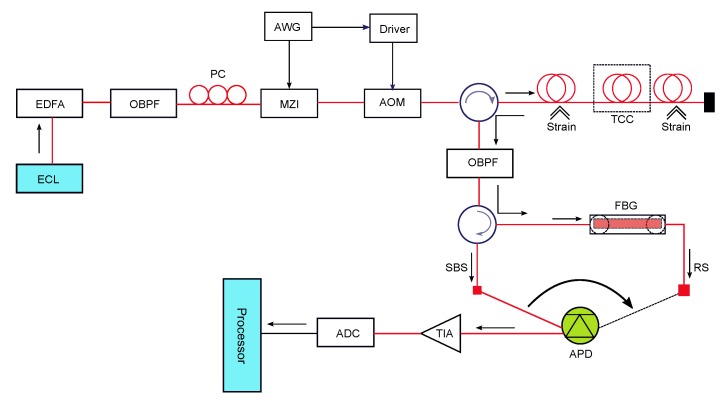
Experimental setup of the Stimulated Brillouin scattering (SBS) implementation of the BDTS system. OF: Optical Filter, PC: Polarization Controller, MZI: Mach–Zehnder Interferometer, BPF: Band-Pass Filter, SBS: Stimulated Brillouin Scattering, WFG: Waveform Generator, ADC: Analog-to-Digital Converter, RS: Rayleigh Scattering, FBG: Fiber–Bragg Grating, ECL: External Cavity Laser, EDFA: Erbium Doped Fiber Amplifier, APD: Avalanche Photo Detector, and TIA: Transimpedance Amplifier. (Adapted from [37].)

Soto et al. [37] proposed a DTS system based on Brillouin scattering, employing pulse coding to achieve a high signal-to-noise ratio (SNR). They noted that the Brillouin-based DTS (BDTS) system can be implemented by exploiting either spontaneous Brillouin scattering (SpBS) or stimulated Brillouin scattering (SBS). SpBS-based BDTS systems are simpler to implement while the SBS-based BDTS systems allow higher SNR and measurement accuracy. The pulse coding scheme used in Soto’s BDTS system can be applied in the Rayleigh scattering-based direct detection ϕ-OTDR system for vibration measurement. The experimental setup of the BDTS system with pulse coding is shown in Figure 9. In this implementation, a pulse-coded OTDR technique is used with a direct detection receiver block. An external cavity laser (ECL) with narrow linewidth (200 KHz) tuned at 1550 nm is used as the light source. A Mach–Zehnder Interferometer (MZI) and high-speed waveform generator (WFG) are used with optical amplifiers and tunable bandpass filters to generate a coded pulse-train, which is injected into the sensing fiber.

Raman scattering-based DTS systems have found some applications over the past two decades. However, from the perspective of simultaneous temperature and strain measurement potentials, the Brillouin scattering-based systems have the following advantages over other scattering-based systems [38]:

- Standard low attenuation loss singlemode fiber can be used, and thus so can the already existing commercial telecommunication equipment and tools.
- Since information gain is not directly affected by the background thermal activation, the Brillouin scattering can be exploited, leading to greater scattering intensity, and hence higher SNR for the backscattered signal.
- The Brillouin-based methods utilize frequency shifting as opposed to Raman-based methods that are intensity based. So, the Brillouin-based methods are inherently more accurate and stable in the long-term, whereas intensity-based Raman systems suffer from higher sensitivity to drift, and from potential biasing issues caused by any step loss.

In Brillouin-based measurements, both intensity and frequency shift of the scattering are sensitive to both temperature and strain variations. To address this cross-sensitivity issue, various pulse coding schemes at low power levels were proposed and implemented in spontaneous Brillouin scattering-based temperature and strain measuring systems. It has been shown that pulse coding can improve the receiver signal-to-noise ratio (SNR) compared to a single pulse with the same peak power level. This allowed for accurate Brillouin intensity and frequency shift measurements, and reduced the adverse effect due to cross-sensitivity between temperature and strain [39].

The spatial resolution of the Brillouin-scattering-based distributed system is dependent on the pulse width. In general, if the laser pulse width is larger than the Brillouin scattering gain, the backscattered signal will have a broader spectral distribution, resulting from the convolution between the Brillouin gain distribution and the incident pulse spectrum. This can significantly distort the backscattered signal over a wide spectral range, and thus cause poor signal detection [40].

## 4. Simultaneous Vibration and Temperature Measurements

Predicting subsurface geologic formations has been a key objective in many geophysical explorations, especially in the oil and gas industry. Different geophysical methods, such as seismic, electrical resistivity, gravity, magnetic, and magnetotelluric, have been used for decades in exploration of natural resources at various depths. Among these, the seismic method has been effective for oil and gas exploration, both onshore and offshore, especially at deeper depths in comparison to other methods. There is, however, a need for simultaneous measurements of vibration and temperature. Measurement of ground vibrations either on the surface or in the borehole are used to estimate wave velocities and density of structures in the surrounding media, and thus to help predict the presence or absence of potential reservoirs in the locations of interest. Wellbore integrity and reservoir performance are two key aspects in the operation of the wells used for hydrocarbon extraction, geothermal applications and hydraulic fracture operations. Temperature is one of the main physical parameters used for enhanced understanding of the downhole flow (or injection) profile through the wells. Fiber-optic-based distributed temperature sensing (DTS) systems have been used to map the temperature profile in a well in real-time without interrupting normal operations [41].

Over the past two decades, fiber-optic-based distributed vibration and temperature measurements have found applications in the oil and gas, mining, environmental, homeland security, and civil engineering industries. However, separate systems are needed, such as DAS for dynamic vibration and DTS for temperature measurements. As discussed in the previous section, different scattering-based techniques are applied in combination with various laser sources (e.g., DFB, ECL, etc.) to achieve high spatial resolution and longer sensing range. There are many applications, especially in geophysical and mining engineering, where simultaneous measurements of vibration and temperature are essential. However, up until recently, separate, expensive and difficult to install and operate systems were deployed to acquire those physical parameters simultaneously.

Recently, Muanenda et al. [10] have designed and experimentally demonstrated a hybrid distributed fiber-optic acoustic and temperature sensing system through integrating Raman-DTS and Rayleigh ϕ-OTDR techniques. In this system, a linear pulse coding scheme [42] is implemented to ensure inter-pulse coherence and intra-pulse incoherence for the purpose of achieving high SNR, and thus increased spatial resolution and sensing range [9,35]. Very recently, Zhang et al. [9] have proposed and demonstrated a hybrid distributed fiber-optic sensing system for multi-parameter (strain, temperature and vibration) detection based on Brillouin-OTDR (B-OTDR) and Rayleigh ϕ-OTDR using a modulated pulse-train. The two systems took advantage of the Simplex pulse coding with a narrow linewidth laser source for generating pulse-trains of varying intensity and width to catalyze Rayleigh phase-shift and Brillouin frequency-shift, and hence for simultaneous multi-parameter measurements. The experimental setup and implementation of the two systems are discussed in the following subsections.

### 4.1. Raman-OTDR and ϕ-OTDR-Based Hybrid Vibration and Temperature Sensing

**Figure 10:**
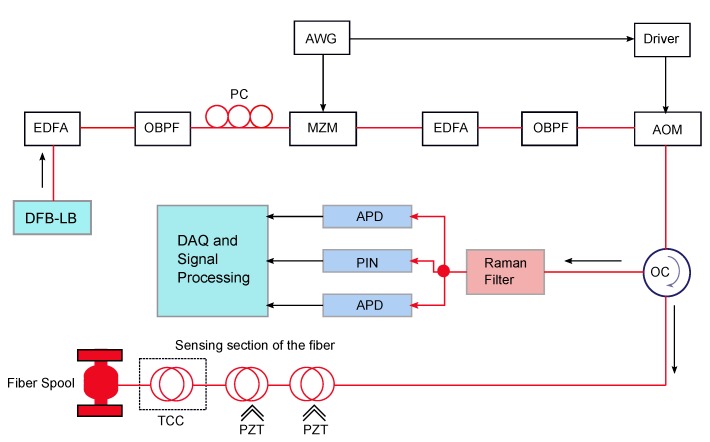
Experimental setup of the hybrid Raman-DTS and ϕ-OTDR system for simultaneous measurements of vibration and temperature. DFB-LD: Distributed Feedback-Laser Diode, EDFA: Erbium Doped Fiber Amplifier, OBPF: Optical Bandpass Filter, MZM: Mach–Zehnder Modulator, WFG: Waveform Generator, AOM: Acousto-optical Modulator, OC: Optical Circulator, APD: Avalanche Photodetector, PC: Polarization Controller. (Adapted from [10].)

Simultaneous measurements of vibration and temperature over the length of an entire measurement distance are a key requirement for monitoring systems, used in the oil and gas industry, hydraulic-fracture-induced microseismic activities, vertical seismic profiling, production flow monitoring, pipeline integrity management and leakage detection. Muanenda et al. [10], for the first time, proposed and experimentally demonstrated a hybrid distributed fiber-optic system for simultaneous measurements of vibration and temperature over a standard singlemode fiber using a commercial off-the-shelf DFB (distributed feedback) laser and common receiver system. That system detected vibrations of up to 500 Hz and temperature with 0.5 ${}^{\circ }$C resolution, both with 5 m spatial resolution and 5 km sensing range. The experimental setup of the hybrid system is shown in Figure 10.

In this implementation, a cyclic Simplex pulse coding is used to modulate pulses that are injected in the sensing fiber. For a standard ϕ-OTDR-based system, linear Simplex coding is used for modulation, where coded traces are obtained as linear superpositions of the backscattered signal intensities of the pulses in the pulse-train. The pulse-train generated using a cyclic Simplex coding scheme for this implementation can be expressed as [35,42]:

(6) $$I_{ij}=I_{i}+I_{j}+2\sqrt{I_{i}I_{j}}\cos\phi_{ij}$$

where $I_{ij}$ is the intensity of the modulated pulse-train, $I_{i}$ and $I_{j}$ are backscattered intensities from two different pulses, and $\phi_{ij}$ is the phase difference between the pulses. The time between two successive pulses in the pulse-train is made to be larger than the coherence time of the laser to ensure that intensities of the backscattered signals are additive, thus, to take advantage of the pulse coding in the ϕ-OTDR technique. Both intra-pulse coherence and inter-pulse incoherence put a limit on the length of the laser linewidth. To use pulse coding with the ϕ-OTDR system, the laser linewidth has to satisfy two interference conditions which are used to determine the upper and lower limit of the linewidth. These conditions are [35]:

(7) $$\Delta v\leq \Delta v_{max}=\frac{1}{\pi \times \tau }$$

where Δv is the laser linewidth, Δ$v_{max}$ is the upper limit of the linewidth, and τ is the pulse width, and

(8) $$\Delta v\geq \Delta v_{min}=\frac{N_{max}}{\pi \times RTT}$$

where Δ$v_{min}$ is the lower limit of the laser linewidth, $N_{max}$ is the maximum codeword length, and RTT is the laser round trip time. The contribution of various pulses within the pulse code to the total intensity in the backscattered signal at the receiver can be calculated through a superposition of the delayed intensity from individual pulses.

**Figure 11:**
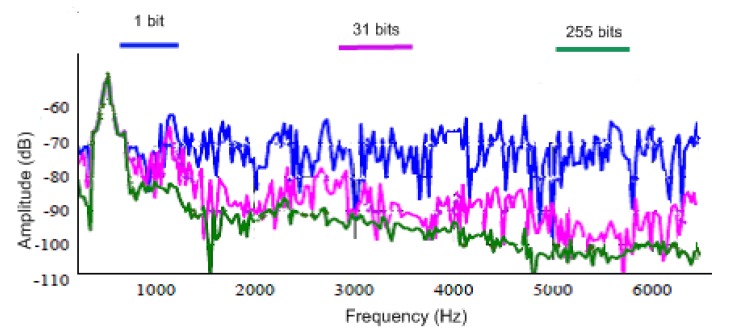
Vibration spectra with different length pulse-trains using cyclic Simplex coding. (Adapted from [10].)

**Figure 12:**
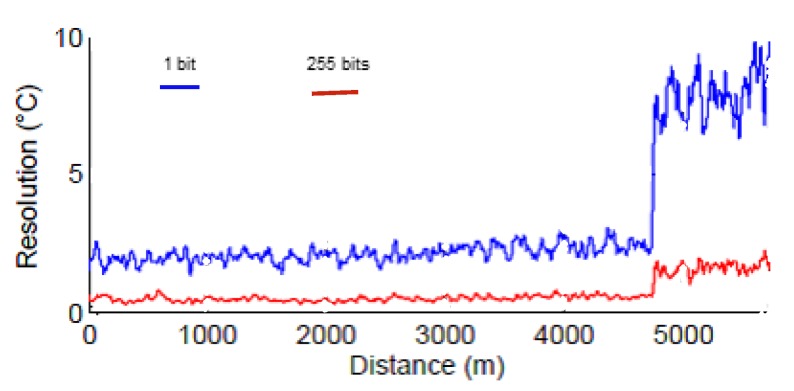
Temperature resolution vs. distance plots with a single pulse and pulse-train. (Adapted from [10].)

For this experiment, a commercial off-the-shelf DFB laser with linewidth range between 1 MHz and 5 MHz at 1549 nm wavelength is used as the light source to ensure intra-pulse coherence and inter-pulse incoherence. Rayleigh-based ϕ-OTDR, and Raman Stoke (S) and Anti-Stoke (AS)-based OTDR, were integrated with a common receiver block, consisting of a high-speed photodiode for Rayleigh detection and two avalanche photodetectors (APD) for Raman Stoke and Anti-stoke detection. For both cases, a direct detection method with a single analog-to-digital converter (ADC) is used for synchronization, and thus simultaneous measurements of vibration and temperature. Power level over the measured frequency band and temperature resolution over distance are plotted and compared for two different length pulse-trains in the Figure 11 and Figure 12, respectively. Higher temperature resolution and lower noise profile are observed for a 255-bit length pulse-train.

### 4.2. B-OTDR and ϕ-OTDR-Based Hybrid Vibration, Temperature and Strain Sensing

Distributed multi-parameter detection using standard telecommunication optical fiber is a very recent development. Zhang et al. [9] demonstrated the feasibility of a hybrid system for simultaneous measurements of dynamic vibration, static temperature and strain along the length of a singlemode optical fiber. In this system, the Brillouin-OTDR and Rayleigh ϕ-OTDR techniques were integrated, a modulated laser pulse-train is injected in the sensing fiber, and the backscattered signals were acquired using a single photodetector (PD) in the direct detection method.

**Figure 13:**
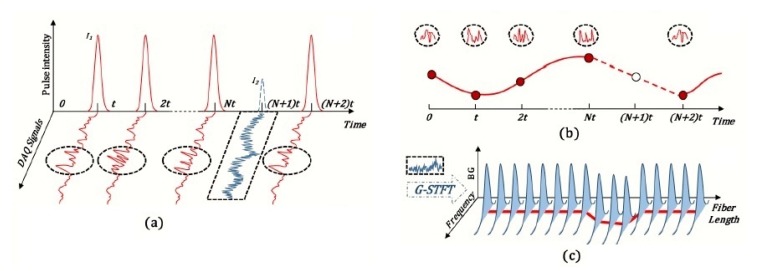
Laser pulse modulation and signal processing of the hybrid distributed multi-parameter sensing system: (**a**) modulated laser sequence and corresponding acquired signal; (**b**) ϕ-OTDR data processing at the vibration point; (**c**) B-OTDR data processing with Gaussian windowed short time Fourier transform (STFT), BG: Brillouin Gain. (Adapted from [9]).

The pulse width and intensity of the laser were modulated using a Simplex coding scheme to improve spatial resolution and achieve simultaneous multi-parameter measurements over a long distance. The modulated pulse-train consists of a group of high-intensity wide pulses and a low-intensity narrow pulse (Figure 13). The time-domain pulses pattern for this setup can be expressed as [9]:

(9) $$y\left(t\right)=\sum_{a=1}^{N}I_{1}\left(aT-t\right)+I_{2}\left(\left(N+1\right)T-t\right)$$

where $I_{1}$ and $I_{2}$ are two pulse profiles that form the pulse-train. A Gaussian profile was chosen to maintain a balance between the pulse power and spatial resolution.

**Figure 14:**
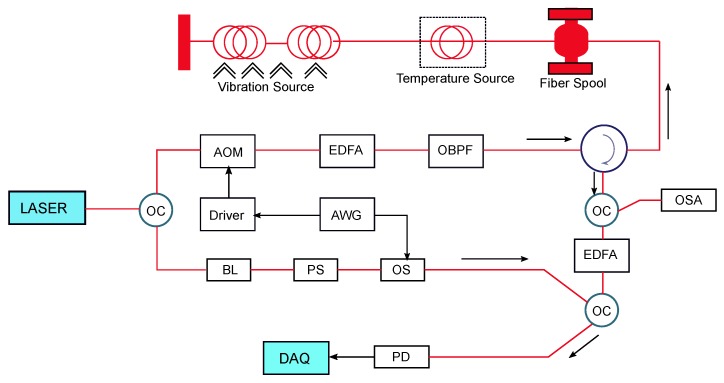
Experimental setup of the distributed simultaneous temperature, vibration and strain sensing system. AOM: Acousto-Optic Modulator, EDFA: Erbium-Doped Fiber Amplifier, BPF: Band-Pass Filter, PZT: Piezoelectric Transducer, AWG: Arbitrary Waveform Generator, BL: Brillouin Laser, PS: Polarization Scrambler, OS: Optical Switch, PD: Photo-Detector, DAQ: Data Acquisition. (Adapted from [9].)

The high-intensity wide pulses are used to provide high optical power enough to activate strong spontaneous Rayleigh scattering, and thus high SNR in the ϕ-OTDR-based system for vibration detection. The low-intensity narrow pulse is used for temperature and strain measurements in the frequency-based B-OTDR system due to its immunity to spurious current noise. A narrow linewidth laser source critical for the ϕ-OTDR-based system is used to ensure coherence in the backscattered light, especially in long sensing fiber. However, a narrow linewidth with high-peak power pulse can stimulate Brillouin scattering (SBS) and give rise to nonlinear effects. The nonlinear effects and SBS can significantly degrade the overall SNR and even make the signal difficult to detect. On the other hand, a low pulse power limits the fiber sensing range due to intrinsic attenuation loss. Pulse width is inversely proportional to spatial resolution. There is a trade-off between length of the pulse-train and pulse power since they both affect the SNR, spatial resolution and sensing range. These parameters can be adjusted for optimum performance in particular applications. The experimental setup and implementation of the hybrid distributed multi-parameter detection system is shown in the Figure 14.

The experimental results show that the multi-parameter system acquired vibration signals of up to 4.8 kHz with 3 m spatial resolution, while the spatial resolution for both temperature and strain was 0.8 m over a 10 km standard singlemode telecommunication fiber.

## 5. Challenges and Limitations of Fiber-Optic Sensing

### 5.1. Fiber Design for Distributed Sensing Technology

Standard telecommunication fibers (either singlemode or multimode) are designed to increase the data transmission rate with minimum loss and distortion. Backscattered light in the fiber is an unwanted phenomenon for most telecommunication applications. In general, improvements of telecommunication optical fiber have been focused on the design of the fiber core to facilitate smooth passage of light with minimum reflection, scattering and attenuation loss. Distributed fiber-optic sensors based on standard telecommunication fiber suffer an intrinsic drawback—it is difficult to distinguish changes in backscatter light that arise from temperature, vibration and strain fluctuations, and those caused by intrinsic fiber loss. Considering this, one potential FOS research topic would be the overall fiber design that takes into account not only the core but also the cladding and protective sheath to maximize sensitivity to environmental change in temperature, vibration and strain. There are active investigations focused on designing optical fiber cable that can operate in extreme environments (high temperature, e.g., $800{\;}^{\circ }$C) [43].

### 5.2. Limitations in Sensing Range, Spatial Resolution, Strain and Temperature Accuracies and Overall Performance

Rayleigh, Brillouin, and Raman scattering-based OTDR sensing systems are capable of measuring multiple parameters (vibration, strain, and temperature) separately over variable sensing length, spatial resolution, and frequency bandwidth with different accuracy and sensitivity. The limitations in sensing range can be attributed to inherent attenuation loss over a long sensing distance. Spatial resolution is inversely related to the length of the pulse pattern deployed in a sensing system. It is shown that a longer pulse-train corresponds to a higher signal-to-noise ratio (SNR), and so at the expense of coarse spatial resolution. Temperature and strain resolutions are largely dependent on the materials used to construct the fiber in addition to source laser linewidth and peak frequency [1]. Based on Bao and Chen [1], we have presented a list (Table 2) of temperature and strain accuracies for various distributed fiber-optic sensors.

The performance summary of various ϕ-OTDR systems in terms of their respective sensing range, spatial resolution and maximum measured frequency are listed in Table 3. The list is based on research systems developed by various academic and research institutions as mentioned in an article by Liu et al. [17].

### 5.3. Challenges of DVTS Systems for Simultaneous Measurements of Vibration and Temperature

Simultaneous measurements of vibration and temperature using the same fiber and the laser source inherently pose limitations since two different schemes such as B-OTDR and ϕ-OTDR are implemented through combining pulses with different intensity and width. For example, high pulse intensity is required for vibration detection using the ϕ-OTDR method to provide enough optical energy to activate Rayleigh scattering in the fiber. On the other hand, the signal-to-noise ratio (SNR) in temperature measurements using the B-OTDR method will suffer with high peak power through the activation of spontaneous Brillouin scattering and other nonlinear effects in the fiber. Also, combining two different pulse patterns can increase the overall pulse width, and thus coarse spatial resolution and lower sample repetition rate. The wider the pulse-train, the higher the likelihood that some backscattered signals will interact with parts of the incoming pulse-train.

## 6. DVTS Systems in Geophysical Applications

Fiber-optic distributed sensing for multi-parameter measurements have been exploited over the past decades in various fields including the oil and gas industry, mining, civil, homeland security and environmental engineering. Increasing demand for fossil fuel has been pushing the technological boundary of gas and oil exploration, and its safe operation. Fiber-optic sensing technology (FOS) has become particularly attractive considering its ability to operate in restricted space, measure multiple parameters over long distance, have low-power requirement, survive harsh environments (high temperature and pressure), and protect itself against potential electromagnetic interference [41,44,45].

### 6.1. Improved Production and Reservoir Monitoring

Frequent, time-lapse measurements of temperature, vibration and strain in and around the wellbore can provide critical reservoir information, used for production and recovery optimization. Currently, multiple fibers are used for distributed measurements using separate DAS, DTS, and DPS (distributed pressure sensing) systems. The separate distributed systems require additional fiber-optic cables, equipment, installations and acquisition of three different large data sets. This adds unnecessary tasks to the overall reservoir management workflows, increases cost, and ultimately limits applications of the technology in future well and reservoir surveillance [46,47].

Hydraulic fracturing monitoring, production profiling for oil and gas producers, injection profiling for water source management, gas lift monitoring and wellbore seismic data acquisition are some applications where an integrated hybrid multi-parameter fiber-optic sensing system can be robust and cost effective [5,48]. Permanently installed multi-parameter fiber-optic sensing systems will enable time-lapse data acquisition surveys without well intervention, in real-time. Repeated avoidance of well intervention will also minimize operational risks and production interruption.

### 6.2. Real-Time Permanent Subsea Wells Monitoring

Operation and maintenance of subsea wells are considered expensive and high risk investments. Hence, implementation of any new technology such as fiber-optic technology in subsea applications is rarely implemented even though added value is supported by subsurface engineering and geoscientists. Surveillance to reduce risks in subsea wells requires information on down-hole flowing and shut-in pressures, buildup or drawdown pressure, time-lapse production profile, flowing and static down-hole temperatures, multiphase flow rate, and water or gas breakthrough [49]. The data types of this information are temperature, pressure and vibration over the well length. These requirements can be met through developing and deploying subsea-specific hybrid fiber-optic sensing systems that are reliable and robust yet low-cost.

Permanently installed fiber-optic distributed sensors give users an option for continuous real-time monitoring without additional installations. Fiber-optic sensing systems have the following advantages over other technologies, especially in borehole measurements: (1) no down-hole electronics which decreases the failure rate, and (2) relative low mass of the sensing element (fiber-optic cable plus supporting structure), which increases equipment vibration tolerance, and thus minimizes noise in the acquired data [50]. Subsea cable installation, fiber-medium coupling in a turbulent and high-pressure underwater environment, data processing techniques and long-term reliability are some technical challenges that need to be addressed before the fiber-optic sensing technology can be commercially deployed.

### 6.3. Mine Safety and Operations

The safety of mining personnel and long-term operations have been the key to successful and economic extraction of natural resources in the subsurface mining environment. Implementation of advanced technology in structural integrity monitoring systems, personal communication devices, and safety equipment and training have drastically cut down total mining fatalities over the past four decades. According to the Mine Safety and Health Association (MSHA),the mining industry experienced 25 fatalities in 2016, which is a record low, compared to 245 in 1978 [51].

Ground deformation, induced seismic activity during explosive charge and movement of heavy equipment, in addition to geological features such as faults and cracks, are the leading causes of structural instability in underground mines. Real-time monitoring of stress, strain and vibration along the entire mine network can provide useful information on the structural integrity, and thus can prevent catastrophic failures and loss of life and personnel injuries. Temperature fluctuations in an underground mine environment directly affect the health and safety of mining personnel. Major safety issues in underground mines can be due to a rise in temperature, resulting for example from explosive blasting. Early detection and localization of temperature variations along the entire mine network is considered as a critical indicator of potential hazards. The temperature profile obtained from real-time and continuous measurement can assist in the mine ventilation condition assessment, and possible rerouting plans, in case of hazardous situations [52,53].

Point sensors and recently quasi-distributed sensing systems have been the state-of-the-art in detecting and localizing ground deformation in discrete intervals at strategic locations inside the mine. These sensing devices and associated control systems are electro-mechanical, and prone to failure in harsh environments (high temperature and pressure) either during normal operations and/or due to structural collapse. Also, devices for communication to/from the control module require additional equipment, including a cable network along the mine and power supply, which are vulnerable to disruption due to tear and breakdown caused by structural collapse. In those scenarios, a hybrid fiber-optic distributed vibration and temperature sensing (DVTS) system can be used for real-time and continuous measurements of temperature and dynamic vibration along the entire length of the mine with fine spatial resolution. The DVTS systems are resilient to harsh operating environments and can withstand structural failure with embedded real-time communication capability to/from the surface command and control center. Thus, they can provide uninterrupted stress and temperature information along the length of the sensing optical fiber, with high accuracy in terms of event location, and alert the mine operators to take any preventive measures.

### 6.4. Leak Detection in Pipeline and Abandoned Gas Wells

Pipelines carrying oil or gas, and abandoned wells often cross hazardous environmental areas, such as landslides and earthquakes, and can be subject to vandalism and obstruction. These hazards and adverse human activities can lead to damage in the structural integrity of the pipeline and well sealing, and to subsequent catastrophic failure. Leakage of toxic gas and oils from pipelines and abandoned wells can have a catastrophic effect on the environment, ecosystem, marine and wildlife habitats. Distributed FOS technology has the ability to measure vibration, temperature, and strain at thousands of points along a single fiber. Recent developments in DAS, DTS and DVTS technologies promise to provide cost-effective tools allowing monitoring critical parameters over tens of kilometers along the pipeline with a single distributed system [7,54,55].

Pipelines deployed in a subfreezing environment (e.g., Arctic and Antarctic) are designed not to leak under normal operating conditions. However, excessive strain and temperature fluctuations triggered by ice gouging, strudel scour, frost heave and permafrost thaw along with other extreme loading and corrosions, could result in leaks. These leaks cause local anomalies in temperature and vibration signatures, which can be detected and monitored regularly using distributed sensing systems [54].

Before any oil or gas well is completely abandoned either for business or technical purpose, the well must be isolated and all porous zones be covered to prevent unauthorized production and leaks. Monitoring changes in physical parameters with distributed FOS systems allows for leak detection within the well-bore and fluid migration mapping through the casing with full-wellbore coverage. An integrated DAS and DTS system can accurately determine location and number of leaks, dynamic movement of fluid migration caused due to failures in casings, wellhead seals and control valves [7]. This information can be archived into a database to characterize different leak types and their relation to frequency and amplitude variations of temperature, flow rate, strain and vibration. This can be a useful tool for understanding and preventing future catastrophic failures and leaks.

### 6.5. Induced Microseismic Monitoring

Development of sophisticated technologies made shale gas and tight oil exploration and production feasible, despite the recent downturn in the global oil prices. The core technology behind this type of production is called hydraulic fracturing, where a mix of water, sand and chemicals is injected into the ground to break apart the rock and release the trapped oil and gas. Several studies [56,57] have inferred a correlation between local earthquakes due to induced microseismic activities and hydraulic fracturing in the vicinity. A distributed acoustic sensing (DAS) system deployed in the wellbore can detect both magnitude and locations of micro-seismic events in the nearby area, which can be useful for oil and gas production optimization as well as predicting potential earthquakes [58].

Fiber-optic distributed vibration systems can be deployed both on the surface and in the borehole to collect vibration data during and after hydraulic fracturing operations. These data can then be analyzed using existing earthquake prediction methods for predicting any potential induced earthquakes in the vicinity. Integrated DAS and DTS systems can also be deployed in underground mines to monitor the local environment, stress-induced rock shifts, and micro-seismic events to ensure mine safety and operations [59].

### 6.6. Certain Geohazards Monitoring

Landslides, soil erosion, levee collapses and ground subsidence constitute a significant percentage of modern day recurring geohazards [60]. Time-lapse monitoring of soil levees and embankments can be useful in preventing the collapse of the supporting structure. Local and distributed measurements of temperature inside the levee are recognized as an important tool in identifying water-flow fluctuations across the levee. It is shown that temperature measurements using distributed temperature sensing (DTS) systems can be used for leakage detection in levees [61,62]. Strain field variations within the soil levees and embankments are another useful parameter for detecting any potential structural collapse. Real-time monitoring of small displacements with fine spatial resolution and high sensitivity is a key assessment tool for preventing any potential soil levee- and embankment-related geohazard [60]. Traditionally, two separate DSS (distributed strain sensing) and DTS systems are used for strain and temperature measurement. For those applications, a DVTS system can potentially be used to measure both the strain and temperature simultaneously without the need for two separate systems.

### 6.7. Geothermal Energy Exploration and Production Monitoring

Geothermal energy is the heat trapped in the Earth and is available either as hot water or steam [63]. The local temperature gradient (geothermal gradient) with depth in the Earth is used for the qualitative assessment of a potential geothermal site. Temperature and vibration measurements are used to identify geologic features associated with geothermal energy and the temperature gradient through the structure. Sites with a high temperature gradient at shallow depth are considered economically viable for geothermal energy production. Keeping this in mind, a distributed fiber-optic sensing system with a simultaneous dynamic vibration and temperature measurement capability can be useful during both exploration and production monitoring.

## 7. Challenges Associated with Geophysical Applications

### 7.1. Fiber–Medium Coupling

In conventional land-based seismic surveys, the geophones are either planted on the ground for surface seismic profiling (SSP) or mounted inside the borehole for vertical seismic profiling (VSP). However, for distributed fiber-optic systems, coupling between the cable and medium remains a great challenge. In particular, for vibration measurements, an efficient transfer of source energy to the receiver (optical fiber) is critical, and thus requires strong coupling between the cable and medium [64]. Typical coupling techniques implemented for DAS and DTS in VSP applications include permanently cementing the cable behind well casing, clamping the cable to production tubing inside the casing, and a wireline deployment where the cable is installed loose inside the borehole. Pros and cons of these techniques are characterized in terms of relative data quality, deployment complexity and reusability. Out of the three techniques, permanently cementing the cable behind the casing provided the highest data quality and was simpler to deploy, but can only be used in a particular borehole [45,65].

**Figure 15:**
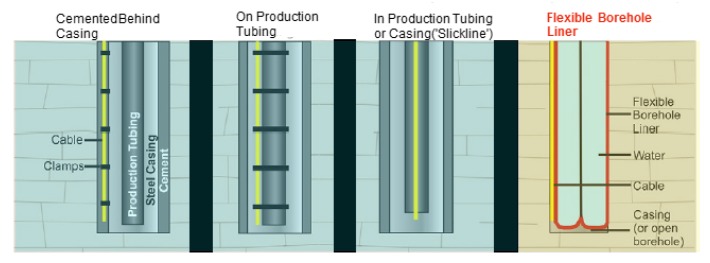
Schematic displaying coupling between the sensing cable and medium for the three conventional and the new flexible liner techniques used for measurements in the borehole. Relative data quality, cost, removability and effective operational depth are compared for these techniques. (Adapted from [64]).

To address this limitation, Munn et al. [64] proposed and implemented a novel coupling solution using flexible borehole liners to couple the fiber-optic cable against the borehole wall for shallow DAS VSP surveys. Comparisons among the three conventional coupling techniques and the new technique are depicted in Figure 15. It is concluded that the flexible borehole liner-based coupling offers a low-cost option with high data quality, and is easy to deploy and remove at the end of the survey. However, the main disadvantage of this technique over the others is depth limitation (approximately 425 m).

The method discussed here to improve sensor–medium coupling in the borehole using a flexible liner for VSP surveys can also be used for other similar applications, including measuring reservoir fluid flow, monitoring microseismic activities in/around the wellbore during and after hydraulic fracturing. A borehole liner made with vibration sensitive materials can improve overall data quality through a higher signal-to-noise ratio (SNR).

**Figure 16:**
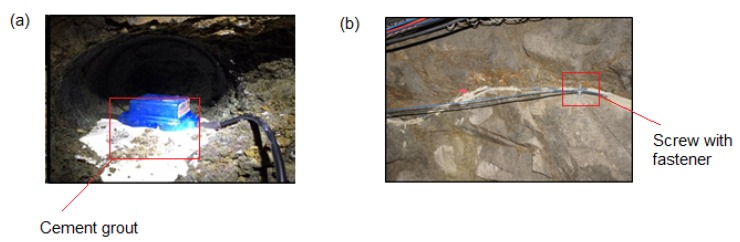
(**a**) Cement-based grout is used to couple a single-component geophone with the rock, and (**b**) the fiber-optic cables are coupled with the mine wall using a metal screw mounted fastener [66].

Geologic stability and temperature variations are major concerns in all underground mines, since they have a direct bearing on safety and personnel. In a recent investigation at Montana Tech of the University of Montana [66], a commercially available fiber-optic distributed acoustic sensing (DAS) system was used to measure dynamic strain caused by ground vibration along various segments of an underground mine, and then compared with conventional electromechanical geophone-based measurements. The main objective of the investigation was to assess the fiber–medium coupling in the hardrock mining environment. Fiber-optic cables were coupled with the mine walls (Figure 16) using metal screws and fasteners at discrete locations in the mine. Comparisons were made between the fiber-optic DAS and the reference geophone-based vibration signals. It was observed that the metal screw/fastener combination provided better fiber–rock coupling, and thus increased signal fidelity compared to areas where the cables were barely in direct contact (without any fastener) with the mine wall.

**Figure 17:**
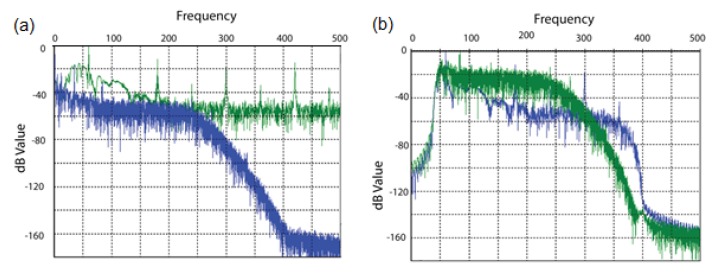
Comparison of vibration signals between (**a**) the reference geophone (blue) and the fiber-optic cable (green); and (**b**) the reference geophone (blue) and the buried (approximately 15 cm under the surface) fiber-optic cable (green) [66].

Distributed fiber-optic sensing for surface seismic profiling (SSP) in the oil and gas industry is very limited due to these main factors: (1) poor coupling between the sensing cable and the ground; (2) limited frequency bandwidth; and (3) single component data, unlike multi-component data from traditional geophones. Burying the optical cable in a shallow trench can provide better coupling and thus higher SNR than just laying it on the ground for some SSP applications (Figure 17). However, it is impractical to dig a trench over a long distance on difficult terrains.

### 7.2. Multi-Component Distributed Strain Sensing

One of the main limitations of using fiber-optic distributed sensing for strain or vibration measurement is lack of directionality. The standard DAS (distributed acoustic sensing) systems are more sensitive in the axial direction compared to the radial direction. Thus, the systems are capable of measuring strain with an acceptable signal-to-noise ratio (SNR) only in the axial direction [67]. In conventional multi-component geophone-based measurements, both axial and radial components of strain can be measured. Recently, Lim et al. [68] proposed two novel methods to obtain multi-component DAS data using different optical fiber configurations. One of the methods uses multiple parallel fibers and is based on axial gradient measurements to obtain curvature of the fibers. This information is then used to reconstruct the multiple components of displacement around the fibers. The second method uses a single optical fiber in a helical arrangement to obtain angle-dependent strains, which are used to reconstruct the strain tensor of the surrounding field. The study concluded that either multiple parallel fibers or a single helical fiber can be used to simulate multi-component fiber-optic DAS data [68]. This recent development has the potential for broad geophysical applications of the DVTS systems, especially for multi-component surface seismic profile (SSP) and vertical seismic profile (VSP) measurements, in addition to temperature measurements.

## 8. Conclusions

Fiber-optic sensing technology has revolutionized distributed multi-parameter measurements across many sectors with its novel applications. The oil and gas industry has emerged as one of the key players in advancing the distributed sensing technology for energy exploration, monitoring reservoir integrity, and production optimization. Recent development in hybrid systems, capable of simultaneous measurements of vibration and temperature, is a game-changer, considering performance improvement and low cost in comparison to single parameter measurement systems. However, poor fiber–medium coupling, low sensitivity of standard optical fibers to external temperature and vibration, and large-scale data processing for real-time applications, are major limitations in achieving maturity and reliability of the distributed hybrid sensing technology. In this paper, we presented the latest developments in different fiber-optic sensing technology focused on multi-parameter measurements, and their applications in geophysical engineering, including the limitations and opportunities for research and development. 

## Figures and Tables

**Table 1:** Attenuation loss of different types of optical fiber. (Adapted from [13].)

Fiber Type		Singlemode	Multimode	Multimode
Cladding Diameter		125 μm	125 μm	125 μm
Core Diameter		8–10 μm	50 μm	62.5 μ m
Attenuation (dB/km)	850 nm	N/A	2.5	3.5
	1300/1310 nm	0.3	0.8	1.4
	1550 nm	0.2	N/A	N/A

**Table 2:** Strain and Temperature Accuracy. (Adapted from [1].)

	Brillouin-OTDR	Raman-OTDR	Rayleigh-OFDR	Rayleigh ϕ-OTDR
Strain and Temperature?	Yes	No	Yes	No
Strain Accuracy (μϵ)	60	N/A	1	N/A
Temperature Accuracy (${}^{\circ }$C)	2–3	0.8	0.1	N/A

**Table 3:** Performance chart of various ϕ-OTDR-based systems. (Adapted from [17].)

Sensing Range (km)	Spatial Resolution (m)	Frequency? Yes/No (Hz)
19	200	No
1	0.5	8 k
1.25	5	39.5 k
125	10	250
131.5	8	373
175	25	Yes
44	5	No
9	2	1 k
1.064	5	3 M
1.150	5	6.3 M
1	2	5 k
10	6	Yes
